# Improving outcomes for ill and injured children in emergency departments: protocol for a program in pediatric emergency medicine and knowledge translation science

**DOI:** 10.1186/1748-5908-4-60

**Published:** 2009-09-22

**Authors:** Shannon Scott, Lisa Hartling, Jeremy Grimshaw, David Johnson, Martin Osmond, Amy Plint, Rollin Brant, Jamie C Brehaut, Ian D Graham, Gillian Currie, Nicola Shaw, Maala Bhatt, Tim Lynch, Liza Bialy, Terry Klassen

**Affiliations:** 1 Faculty of Nursing, University of Alberta, Edmonton, Canada; 2 Department of Pediatrics, Faculty of Medicine and Dentistry, University of Alberta, Edmonton, Canada; 3 Ottawa Health Research Institute, Faculty of Medicine, University of Ottawa, Ottawa, Canada; 4 Department of Pediatrics, Faculty of Medicine, University of Calgary, Calgary, Canada; 5 Departments of Pediatric and Emergency Medicine, University of Ottawa, Ottawa, Canada; 6 Department of Statistics, University of British Columbia, Vancouver, Canada; 7 Canadian Institutes of Health Research, Ottawa, Canada; 8 Department of Family Medicine, Faculty of Medicine and Dentistry, University of Alberta, Edmonton, Canada; 9 Division of Emergency Medicine, Faculty of Medicine, McGill University, Montreal, Canada; 10 Department of Pediatrics, Faculty of Medicine, University of Western Ontario, London, Canada

## Abstract

Approximately one-quarter of all Canadian children will seek emergency care in any given year, with the two most common medical problems affecting children in the emergency department (ED) being acute respiratory illness and injury. Treatment for some medical conditions in the ED remains controversial due to a lack of strong supporting evidence.

The purpose of this paper is to describe a multi-centre team grant in pediatric emergency medicine (PEM) that has been recently funded by the Canadian Institutes of Health Research (CIHR). This program of research integrates clinical research (in the areas of acute respiratory illness and injury) and knowledge translation (KT). This initiative includes seven distinct projects that address the objective to generate new evidence for clinical care and KT in the pediatric ED. Five of the seven research projects in this team grant make significant contributions to knowledge development in KT science, and these contributions are the focus of this paper.

The research designs employed in this program include: cross-sectional surveys, randomized controlled trials (RCTs), quasi-experimental designs with interrupted time-series analysis and staggered implementation strategies, and qualitative designs.

This team grant provides unique opportunities for making important KT methodological developments, with a particular focus on developing a better theoretical understanding of the causal mechanisms and effect modifiers of different KT interventions.

## Background

In any given year, almost one-quarter of Canadian children seek emergency care and approximately 6% of these visits result in a hospital stay. In 2000, there were approximately 400,000 visits to Canadian emergency departments (ED) housed in children's hospitals, representing an annual rate of 534 pediatric emergency department (PED) visits per 10,000 children [1]. The research program described here focuses on the two most common medical problems affecting children in the ED: acute respiratory illness and injury. Despite the magnitude and severity of these problems, the ED care for some conditions remains controversial due to a lack of strong evidence and in many cases where evidence does exist medical practice has been slow to change.

### Research objectives

The overall goal of the team grant is to improve the health outcomes of children with respiratory distress and injury presenting to the ED. The primary objectives of the Team Grant are to: generate new evidence for clinical care and knowledge translation (KT) through the conduct of rigorous multi-centered studies; train and mentor students, fellows, and new researchers in order to increase clinician and researcher capacity; and develop and advance a research agenda for methodological issues that arise during the conduct of the component research projects.

### Research Framework

**Figure 1:**
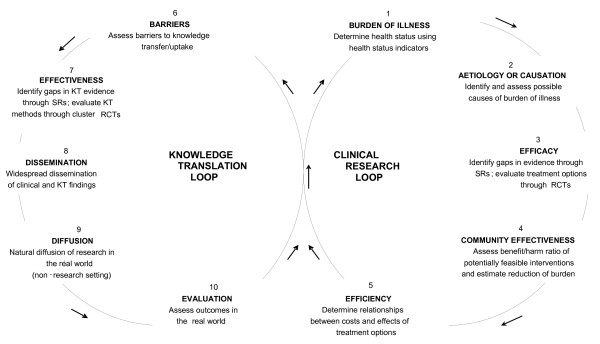
**The iterative figure-eight - A pictorial representation of a framework for the collection, utilization, and evaluation of health information**.

The theoretical model for the research program is based on the figure eight iterative loop [2] [see Figure 1]. This a modified version of an original 'measurement iterative loop' first suggested by Tugwell and colleagues [3] in 1985 and illustrated in Hartling and colleagues [2]. Similar approaches using an iterative loop have previously been used in other clinical research areas to provide a framework for the planning, implementation, and evaluation of clinical strategies to reduce the burden of illness in specific clinical areas. The move to adopt a figure eight iterative loop arose when we became aware of the gap between 'what we know and what we do', and the need to address KT strategies as they relate to clinical research [2]. The 'figure eight' shape is comprised of two loops, a clinical research loop and a KT loop, which together provide a framework for collecting, utilizing, and evaluating health information. The iterative loop addresses the need to assess the effectiveness of clinical strategies arising from clinical research as they are implemented in real-world situations.

Adding the KT loop to the model introduces an important complexity. That is, in the KT loop not only are clinical research findings being implemented, we are also developing new knowledge through assessing strategies that are effective to implementing the research. The KT loop addresses the need to disseminate the research findings from both clinical (for practitioners) and KT research (for researchers), and to assess their outcomes and effectiveness in the real world of clinical practice [2]. The loop demonstrates how evaluation in the KT loop can lead back to an examination of the burden of illness, the first step in the clinical research loop.

### Component research projects

This team grant in pediatric emergency medicine (PEM) includes seven distinct projects that address the objective to generate new evidence for clinical care and KT. The seven projects will be briefly described as they were originally designed, and the contributions for KT science will be highlighted in the five projects that had an explicit KT component. The clinical merit of the seven projects in this team grant have been highlighted in a complementary publication in Academic Emergency Medicine [2].

### Evaluation of an active strategy to implement the Canadian pediatric computed tomography (CT) head rule

Pediatric minor head injury is a very common but potentially serious condition that results in children seeking care in an ED. ED physicians have little guidance in deciding which children require a costly CT scan of the head. Consequently, members of our research team have conducted a series of studies to develop an accurate and reliable clinical decision rule for CT use in children with minor head injury.

We are going to examine the clinical impact and costs of implementing this CT head rule in both pediatric and community EDs. The implementation phase of this project aims to evaluate the effectiveness of an active KT strategy to implement the Canadian assessment of tomography for childhood head injury (CATCH) rule into physician practice in multiple EDs compared to a control strategy that relies upon passive KT strategies.

The proposed study design is a matched-pair cluster design study which compares outcome measures during three consecutive 12-month 'before', 'after', and 'decay' periods at six pairs of 'intervention' and 'control' sites (200 patients per hospital). We intend to pursue simple and inexpensive strategies to actively implement the use of the CATCH rule at the intervention sites. This includes educational initiatives, mandatory online reminders, and attaining physician agreement in ordering CT scans by the CATCH rule. Proposed primary and secondary outcomes include CT head imaging rates, number of missed neurological intervention cases, patient satisfaction, accuracy of the rule, and physician comfort and compliance.

Implementation of the rule has the potential to significantly reduce health care costs and improve the efficiency and safety of patient care in EDs throughout Canada. Findings from this project will contribute to the area of KT by increasing the understanding on the potential for new knowledge in PEM to be implemented through simple and inexpensive KT measures.

### Management of bronchiolitis in the community ED

Bronchiolitis is the most common disease of the lower respiratory track during the first year of life [4,5]. In Canada, 35 per 1000 children less than one year of age are hospitalized annually with bronchiolitis [6], and a conservative estimate is that it costs over 23 million dollars per year [7]. The goals of this study are: to describe the management of bronchiolitis in community EDs in Ontario; to establish a network of hospitals willing to participate in a cluster randomized trial of different KT strategies for dissemination of a bronchiolitis clinical practice guidelines (CPGs) in community EDs; and to identify potential barriers to implementation of a bronchiolitis CPG within these community EDs. The proposed study design includes a retrospective cohort of consecutive children presenting to community EDs with bronchiolitis over two bronchiolitis seasons, and a semi-structured interview of the medical and/or operation director of each ED. All community-based hospitals throughout Ontario that have 24-hour EDs and accept pediatric patients to the ED and hospital wards will be eligible for the study.

The primary outcome will be the proportion of infants ≤12 months of age diagnosed with bronchiolitis in the ED who are admitted to hospital. Secondary outcomes include clinical presentation (oxygen saturation, respiratory rate, heart rate, and blood pressure), medication usage, and investigations (x-rays, blood tests such as CBC, and viral studies). This will allow us to better tailor proposed KT strategies for use in the community ED. Given the high burden of disease of bronchiolitis, its seasonal nature, the extensive practice variation in treating and investigating the illness, as well as the high admission rates in community hospitals, an effective KT strategy for a bronchiolitis CPG could result in significant savings of healthcare dollars.

### A KT 'laboratory': the development and implementation of clinical pathways for two common childhood emergencies

KT research has shown a persistent gap between what we know and what we actually do [8]. Despite the heightened interest in clinical guidelines, there remains much uncertainty about how best to introduce them into routine practice [9-11], partially due to the varying effectiveness of interventions across different clinical problems, contexts, and organizations. Consequently, we propose using qualitative and theory-based process evaluation to explore the causal mechanisms of implementing clinical pathways (multi-disciplinary plans incorporating best clinical practice for specified groups of patients with a particular diagnosis) for the management of the two most common childhood emergencies, asthma and gastroenteritis.

The primary objective is to evaluate the effectiveness of two clinical pathways by assessing if there is a change in: hospital days per disease episode; the use of known effective therapies; total societal costs; clinical outcomes, especially those with severe outcomes; and families' social and economic burden. A secondary objective is to explore causal mechanisms for how various interventions work by performing qualitative analyses to provide new insights and to generate hypotheses and testing a range of theory-based process evaluations.

This is a quasi-experimental research design that incorporates interrupted time-series analysis and staggered implementation strategies [12,13], as well as the use of interviews (both individual and focus group) and surveys to explore the causal mechanisms that shape the implementation process. Each of the clinical pathways will be developed using accepted standards [14-16], and include a committee broadly representative of all health care professionals and levels of care within the Calgary health region. We will implement the pathways using methods that have been shown to be the most consistently effective, including small group interactive teaching sessions, educational outreach visits for selected key health care professionals, and reminders (standing order sets) in the form of an optimized electronic order entry system (Sunrise™ Clinical Manager, Eclipsys Corporation^®^) [9-11,17].

The primary outcome of our quantitative analysis will be hospital days per disease episode, and secondary outcomes will be the use of known effective therapies for the treatment and management of asthma and gastroenteritis. Outcomes will be assessed using time series analysis. For the qualitative portion of the project, trained investigators will interview clinicians using both focus group and individual interviews in all 12 hospitals before and after implementation of each pathway. Data analysis will be guided by the Ottawa Model of Research Use [18,19]. Data analysis will follow the constant comparative approach [20,21]. The phases of inductive analysis will involve: coding (attributing key words or phrases to salient excerpts of narrative data); categorization of codes based on relationships to each other and the phenomenon of interest; and the development of 'emerging themes'.

We anticipate that through these approaches we will begin to 'look inside the black box' and understand why KT interventions work in some contexts and not others. Potentially, this project aims to have the most significant contribution to KT science, as attributes of both the pathway and implementation context deemed important for successful implementation will be identified. Furthermore, elements that hinder implementation will also be identified--this knowledge is important for future implementation initiatives.

### A qualitative study of barriers and supports to implementation of metered dose inhalers (MDI)/spacer use in Canadian EDs

Acute asthma exacerbation is one of the most common conditions for children presenting to an ED with two main methods of delivering conventional treatment via nebulizer or MDI/spacer being available. It is well established that MDI Spacers are an effective approach to delivering therapeutic treatment resulting in shorter ED stays and fewer patient side effects [22], yet the uptake of this innovation has been slow and haphazard. The adoption of new innovations in health is not easily reduced to the decision of an ED physician, but rather is shaped by contextual factors, perceptions of the innovation, and characteristics of the adopters [23].

The objective of this qualitative case study design project is to understand the system, organizational, and individual factors that shape the implementation of MDI/spacer use in Canadian EDs. Overall study objectives are to determine barriers and supports to implementing MDI/spacer research into PED practice and to identify factors associated with early and late adoption of MDI/spacers in PEDs in Canada. Pediatric hospital EDs will be classified into three groups based on stage of implementation. Those currently using MDI/spacers will be considered 'early adopters' of the innovation and will be compared with EDs currently in the process of adopting MDI/spacers and those EDs that are 'later adopters' of the innovation (no current plans to adopt) [24]. We will purposefully sample [25] three early adopter EDs, three EDs in the process of adopting MDI/spacers, and three late adopter hospitals for comparison.

Employing a comparative case study design, data will be collected via semi-structured in-person interviews and focus groups with the following professionals: a focus group of emergency physicians; a focus group of emergency nurses and respiratory therapists; the chief medical officer of the ED; and the chief nursing officer of the ED. Data collection and analysis will proceed concurrently. Data analysis will be conducted following the constant comparative approach [20,26]. Initial coding will be based on the Ottawa Model of Research Use categories of elements known to influence the uptake of innovations [18,19].

Through this project, we will gain developing knowledge on the key factors at the level of the adopter, innovation, and adopting context that shaped why some institutions were 'early adopters' of the research-based MDI/spacer and why other institutions have yet to adopt it. This knowledge can facilitate future work in optimizing clinical environments for research use.

### Storytelling as a communication tool aimed at parents and children presenting to the ED with common disease conditions

Evidence-based medicine has been heralded as the model for practicing medicine, yet consistently others have argued for a more holistic approach that melds both intuition and anecdotal evidence with research [27]. Storytelling and the use of narratives may be a way of uniting these divergent approaches to produce a dissemination tool that effectively addresses the information needs of parents.

The aim of this project is to integrate the art of storytelling with the science of evidence-based medicine to deliver medical information to parents with a child in the ED. We have developed three 'croup' storybooks, each based on a different level of severity: mild, moderate, or severe. The proposed study design is a randomized controlled trial with hospitals EDs being randomly placed in either 'standard care' or interventions (storybook) groups. Primary outcomes will be parental anxiety assessed using a seven-point Likert scale ranging from extremely stressed to extreme calm. The primary outcome will be change in parental anxiety from baseline (immediately following recruitment to the study at the beginning of the ED visit) to discharge from the ED. For self-reported anxiety, a change score from baseline to discharge will be calculated for each patient. The mean change scores will be compared between groups using an independent-groups t-test if they are normally distributed or the Mann-Whitney test if they are skewed.

Secondary outcomes will include: parental satisfaction with the overall ED visit and the information received in the ED; parental comfort with the information and the ED visit; parental knowledge regarding the natural history of the disease; symptoms and management strategies; parental decisional conflict or regret; ongoing symptoms; healthcare utilization patterns; and costs to healthcare system (based on access to healthcare and prescription medication). Data on secondary outcomes will be gathered through a telephone interview conducted one to ten days following the patients initial visit to the ED

The overall outcome of this research will be to develop consumer-friendly communication tools that have a measurable effect on parental stress, patient outcomes, and health care utilization. This research will inform the development of evidence-based stories in other clinical areas and potentially will develop new knowledge on the potential effectiveness of storytelling as a KT intervention for transfer of research to parents.

### Safety surveillance

Each year, tens of thousands of children receive procedural sedation and analgesia (PSA) for diagnostic and therapeutic procedures in Canadian PEDs. The extremely low incidence rate for severe adverse events has made it difficult for single centre studies to establish evidence-based safe practices for PSA. This multi-centre project will have a direct impact on the care of injured children by providing evidence about the safety of PSA in the PED. It will also serve as a model for the surveillance of other treatments that may have rare but serious adverse events. The objective of this study is to establish prospective surveillance for adverse outcomes in all patients undergoing procedural sedation in PEDs across Canada.

For this study, we will employ a prospective cohort design. All children undergoing PSA for painful procedures at each site will have a standardized electronic data collection form (to be implemented as the local clinical record) completed by the nurse and physician caring for the patient. Information on the pre-procedural state, conditions and circumstances of the sedation, recovery period, and patient demographics will be collected. Two populations of patients will be contacted following discharge from the ED: all patients who experience adverse events will be contacted within one week of their event to obtain further details about the event and their current state of health; and a random sample of all patients will be contacted two weeks following discharge to determine their state of physical and psychological health after receiving PSA in the ED. It is anticipated that 50,000 to 70,000 children will be enrolled in the database after five years. This large cohort will yield substantially more precise estimates of association between rare adverse outcomes and specific sedation practices than are currently published.

The primary outcomes are to document the incidence of minor and major adverse events in ED PSA, to determine the risk factors for adverse events in PSA, and to determine the impact of pre-procedural fasting state on the incidence of adverse events. Secondary outcome measures are: to document the incidence of inadequate sedations as a measure of the efficacy of PSA; to determine the safety of medications or combinations of medications; to determine the safety of pre-procedural narcotic analgesia; to determine best practice for administering procedural sedation; and to assess the impact of PSA on physical and psychological health.

### Exploring novel methods to improve our diagnostic accuracy of childhood bacterial pneumonia

In North America, pneumonia is an important source of childhood morbidity and occasionally mortality [28-32]. In children under five years of age, the annual incidence rates of 30 to 45 per 1,000 children are higher than any other time of life, except for the elderly [29-31,33-35].

This study will explore whether high-resolution nuclear medicine resonance spectroscopy and an optimal combination of clinical findings determined by expert panels, is more accurate relative to clinicians when it comes to distinguishing bacterial versus non-bacterial pneumonia in children. The objective of this study is to describe the occurrence of pneumonia and its etiology in a prospective cohort of children presenting to Canadian PEDs with suspected pneumonia.

The proposed study design is a prospective cohort, with a proposed sample size of 250 patients. The accuracy of clinicians in their diagnoses of bacterial pneumonia with respect to our novel reference standard will be measured. The optimal combination of clinical findings along with a small number of laboratory investigations as a prediction rule will be determined. The sensitivity, specificity, and positive and negative predictive values of the clinicians will be calculated. Agreement between experts will be summarized using conventional measures of association, including Cohen's kappa, McNemar's Test, and paired t-tests.

To date a comprehensive literature review has been conducted to categorize reference standards used to assess the accuracy of diagnosing bacterial pneumonia, and to determine what factors are the most reliable for diagnosing bacterial pneumonia given the gold standard used. The practice variation study data have been collected for 2 out of 4 weeks from a total of 11 sites. The prospective portion of this project has been initiated in seven participating sites across Canada.

### Contributions from the team grant to KT Science

KT research in emergency medicine (EM) is in its infancy, in part due to challenges specific to the ED setting. These include: the diversity of cases from minor to major and the diversity of conditions seen; limited continuity of care; management of children across primary and secondary care interfaces; the model of care delivery by teams of health professionals; and the short timeframe and stressful conditions under which diagnoses and decisions are made and treatments are implemented. A comprehensive research program is needed for PEM in order to identify gaps in evidence, generate evidence where it is lacking, evaluate barriers to uptake of evidence, and implement and evaluate mechanisms to augment the application of evidence in clinical care.

Recently, the 2007 Academic Emergency Medicine Consensus Conference was convened in order to develop a KT research agenda and comprehensive approach to KT within the specialty of EM [36]. This event was significant in terms of development of a research agenda in EM. Several members of this research team (TK, IG, JG, DJ, JCB and SS) participated in this venue as members skilled in KT. Some key overriding themes emerged from the conference, including the need for the creation of collaborative KT/EM research networks, and the need to ensure that research teams engage in KT research that is both multidisciplinary and interdisciplinary. We believe that the optimal paradigm for increased and rapid uptake of research evidence is a healthcare program which has embedded within it clinical researchers working synergistically with KT experts, as such, we have designed this program of research in such a fashion to have KT activities embedded within our program of research.

The team will increase capacity for KT through an increased number of relationships with potential partners, as well as a formal collaboration with experts in a research area with specific methodological challenges. Specifically, this program of research will contribute to KT science in terms of: the development and testing of KT strategies for the ED setting; contributions to the theoretical and methodological domains by means of exploring the causal mechanisms for how various interventions work through qualitative analysis; and building KT research capacity through generating a new pool of researchers with KT expertise.

## Competing interests

The authors declare that they have no competing interests.

## Authors' contributions

SS presented the original poster outlining this protocol at an annual Knowledge Utilization colloquium (KU06), contributed to the development of the original proposal, and prepared the majority of this protocol for this venue. LH prepared and wrote the majority of the original grant proposal. JG provided critical feedback in the area of KT. SS, DJ, MO, AP, MB, TL, and TK lead individual projects within this protocol and wrote the majority of their project descriptions. RB contributed to the statistical sections in each of the component projects. JB contributed to the intervention and survey techniques sections in several of the component projects. IG contributed to the KTs sections in several of the component projects. GC contributed to the economic analysis sections in each of the component projects. NS and SL contributed to the data management and information technology sections in several of the component projects. LB assisted in the preparation of the protocol, formatting, and updating.
